# BPA, BPAF and TMBPF Alter Adipogenesis and Fat Accumulation in Human Mesenchymal Stem Cells, with Implications for Obesity

**DOI:** 10.3390/ijms22105363

**Published:** 2021-05-19

**Authors:** Isabel C. Cohen, Emry R. Cohenour, Kristen G. Harnett, Sonya M. Schuh

**Affiliations:** 1 Department of Biology, Saint Mary’s College of California, Moraga, CA 94575, USA; icc3@stmarys-ca.edu (I.C.C.); kgh1@stmarys-ca.edu (K.G.H.); 2 Department of Cell and Molecular Biology, California State University, East Bay, Hayward, CA 94542, USA; emryrc@gmail.com

**Keywords:** BPA, BPAF, TMBPF, endocrine-disrupting chemicals (EDCs), adipogenesis, stem cells, adipose-derived stem cells (ASCs), obesity, food-contact coating, plastics

## Abstract

Bisphenol A (BPA) is an endocrine-disrupting chemical used in the production of plastics, and is linked to developmental, reproductive, and metabolic disorders including obesity. Manufacturers have begun using ‘BPA-free’ alternatives instead of BPA in many consumer products. However, these alternatives have had much less testing and oversight, yet they are already being mass-produced and used across industries from plastics to food-contact coatings. Here, we used human female adipose-derived stem cells (hASCs), a type of adult mesenchymal stem cell, to compare the effects of BPA and BPA alternatives on adipogenesis or fat cell development *in vitro*. We focused on two commonly used BPA replacements, bisphenol AF (BPAF) and tetramethyl bisphenol F (TMBPF; monomer of the new valPure V70 food-contact coating). Human ASCs were differentiated into adipocytes using chemically defined media in the presence of control differentiation media with and without 17β-estradiol (E2; 10 μM), or with increasing doses of BPA (0, 0.1 and 1 μM), BPAF (0, 0.1, 1 and 10 nM), or TMBPF (0, 0.01 and 0.1 μM). After differentiation, the cells were stained and imaged to visualize and quantify the accumulation of lipid vacuoles and number of developing fat cells. Treated cells were also examined for cell viability and apoptosis (programmed cell death) using the respective cellular assays. Similar to E2, BPA at 0.1 μM and BPAF at 0.1 nM, significantly increased adipogenesis and lipid production by 20% compared to control differentiated cells (based on total lipid vacuole number to cell number ratios), whereas higher levels of BPA and BPAF significantly decreased adipogenesis (*p* < 0.005). All tested doses of TMBPF significantly reduced adipogenesis and lipid production by 30–40%, likely at least partially through toxic effects on stem cells, as viable cell numbers decreased and apoptosis levels increased throughout differentiation. These findings indicate that low, environmentally-relevant doses of BPA, BPAF, and TMBPF have significant effects on fat cell development and lipid accumulation, with TMBPF having non-estrogenic, anti-adipogenic effects. These and other recent results may provide a potential cellular mechanism between exposure to bisphenols and human obesity, and underscore the likely impact of these chemicals on fat development in vivo.

## 1. Introduction

Bisphenol A (BPA) is a well-known endocrine-disrupting chemical (EDC) and identified obesogen, which is a chemical that can disrupt or increase normal fat development and lipid metabolism and may cause obesity. BPA has been mass-produced since the 1960s, and is an additive used in epoxy resins and polycarbonate plastics to produce various consumer goods, containers, and equipment [1,2]. Because of its low cost and properties of durability and flexibility when synthesized into polymers, BPA is present in everything from food-contact coatings of metal food and beverage containers, to cosmetic and personal care product packaging, storage containers, thermal receipt paper, electronics, car dashboards, medical and dental devices, tableware, toys, and water supply pipes [1,3,4,5,6,7,8,9,10,11,12]. Thus, exposure to BPA is ubiquitous and unavoidable. The 6 million tons produced per year, and the 8 million tons of plastics that end up in our oceans every year, have severely negative impacts on the environment. BPA’s ability to leach out of products has caused significant amounts to be detected in water and soil systems, along with wastewater from construction and recycling treatment sites [1,4,7,8,13,14,15,16,17,18,19,20,21]. Several previous studies have demonstrated that BPA levels present in these systems are capable of detrimental effects on living organisms, including humans [4,12,15,22,23,24]. Multiple studies report that significant amounts of BPA are present in the global human population’s blood and urine [25,26,27,28,29,30]. The National Health and Nutrition Examination Survey and others have consistently found that approximately 95% of humans have detectable BPA levels in their body fluids [25,28,31].

BPA is a known endocrine disruptor and interferes with normal estrogen signaling by acting as an estrogen mimicker or antagonist and causes hormone-like effects in the body [1,7,12,32,33,34,35]. The adverse health effects of exposure to BPA are far reaching as it has been linked to various hormonal, metabolic, reproductive, and developmental defects. BPA has been implicated in several reproductive disorders including infertility [36,37], reduced sperm count and motility [38], and increased risk for miscarriages and genital birth defects [32,39], as well as other health issues such as asthma and Autism spectrum disorder [20,40,41,42]. Even low-dose exposure to BPA has been associated with increased rates of breast and prostate cancer [43,44,45], brain and behavioral abnormalities, metabolic disorders, and obesity [1,2,3].

Obesity and obesity-related illness including cardiovascular disease are considered one of the most critical current global public health crises, being responsible for even more deaths than the current COVID-19 pandemic [46]. Obesity is associated with environmental, genetic, and socioeconomic factors, however the underlying mechanisms and causes are still not fully understood. Unfortunately, the rates of obesity have been dramatically increasing over the past several decades. It has been found that early-life exposure to BPA and childhood obesity are linked [47]. There is a positive correlation between BPA exposure over time and childhood obesity, but not a significant correlation between consolidated high BPA exposure at a single time point and childhood obesity [48,49]. Obese adolescents ages 16–18 are also more likely to have higher levels of BPA in their urine independent of age, sex, race, education, and physical activity [50], and creatinine-corrected BPA levels tend to be higher in women than in men [51,52]. Higher levels of BPA and BPA analogs in the blood and urine of overweight and obese adults have also been reported [53]. Pregnant mothers exposed to shampoo and cosmetics in plastic containers demonstrate a statistically significant increase in BPA concentrations in their urine 24 h after product use [27]. Further, BPA readily crosses the blood–placental barrier due to its lipophilic structure [54,55], and has been detected in fetal blood, cord blood, breast milk, and amniotic fluid with bioaccumulation in the maternal–fetal–placental unit [56,57]. Furthermore, a recent study reported that eliminating cosmetics and personal care products in plastic containers from women’s daily routines for just three weeks significantly lowers levels of BPA and analogs in their bodies [52]. Lipid-soluble chemicals such as BPA have also been found to accumulate in human adipose (fat) cells and tissue [58,59,60,61]. Taken together, these findings suggest that the frequency and timespan of exposure to BPA can contribute to the cause of childhood and adult obesity, especially in women. Further, with greater use of BPA-containing products including cosmetic, hair, and personal care products, among other exposures, it is likely that most women experience a greater lifetime exposure to BPA and analogs.

Recent studies demonstrate that at the cellular and molecular level, BPA may act as an environmental obesogen, influencing adipogenesis and fat accumulation [2,32,62,63]. Adipose, composed of mature adipocytes, progenitors, and stem cells, is an endocrine tissue and is therefore a target of EDCs. BPA might be linked to obesity through the enhancement of preadipocyte cellular differentiation and the expression of adipogenesis-associated genes and transcription factors. BPA significantly affects adipogenesis in human and rat adipose-derived stem cells in vitro [32,62,63], and acts through an estrogen receptor-mediated pathway affecting the expression of several adipogenic genes [32]. Studies on the effects of BPA on the differentiation of rat and human stem cells into mature adipocytes have reported increased expression of adipogenesis-associated genes, impaired metabolic functioning, increased pro-inflammatory cytokine expression [62], and greater lipid accumulation [63]. Therefore, BPA exposure, especially early in life and acting specifically on adipocyte or mesenchymal stem cells, may have the potential to increase the risk of obesity and obesity-related illnesses, especially for girls and women.

**Figure 1:**
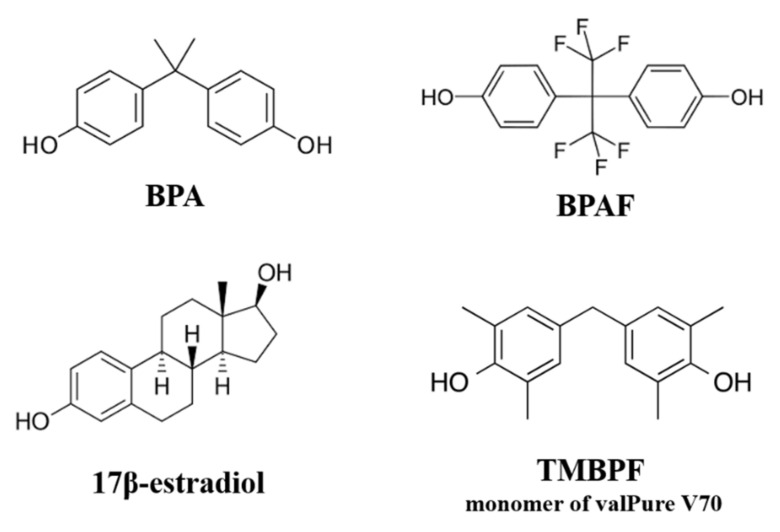
Chemical structures of BPA, BPA alternatives, and 17β-estradiol.

Over three decades of research on the risks of BPA exposure, paired with scientific outreach and public concern demanding BPA-free products, has led to companies producing a next generation of BPA-alternative compounds. These commonly used BPA replacements include bisphenol S (BPS), bisphenol F (BPF), and bisphenol AF (BPAF), which are all quite similar in structure to BPA (Figure 1). Tetramethyl bisphenol F (TMBPF) is one of the most recently used BPA alternatives and is the monomer of the Sherwin-Williams (formerly Valspar) created compound valPure V70, now being used in polymer coatings for the linings of metal beverage and food cans [64]. In a new strategy, TMBPF was selected by using a ‘safety by design’ approach and computational structural analysis to search hundreds of bisphenol chemicals that would share the same BPA-like properties of polymer technical performance, durability, and integrity, but would lack its ability to interfere with estrogen receptors [64]. With very limited independent research performed on TMBPF, a few recent company-sponsored studies conducted in collaboration with academic scientists reported that it lacks the same estrogenic activity and toxicity of BPA and may not have EDC action [65,66,67].

Several recent studies have found that many of the other BPA alternatives including BPS, BPF, and BPAF are not as safe as perceived to be and induce similar or even more potent toxic and estrogenic effects as BPA [68,69,70,71,72,73,74,75,76,77,78,79]. Just like the parent compound BPA, many studies report that these analogs have endocrine-disrupting actions through estrogen agonist and antagonist activity [68], with BPAF being approximately 1,000-fold more potent in its toxic and estrogenic effects than BPA [68,69,71]. Due to BPAF’s chemical structure, where the CH_3_ group of BPA is replaced by a CF_3_ group, it is more electronegative and potentially more reactive. This makes BPAF a more toxic and potent bisphenol. As shown in Figure 1, BPA, BPAF, and TMBPF all possess a very similar lipophilic, phenolic ring structure, allowing them to pass through the cell membrane and various barriers throughout the body (blood–brain, blood–testis, blood–placental–fetal, etc.) [54,55,56,57]. Several studies have found that these BPA alternatives are cytotoxic and lead to apoptosis in rat and human stem cells [71], disrupted embryo development in Zebrafish [68], *Xenopus laevis* [69], and other species, have detrimental effects on reproduction through oogonial, spermatogonial and testicular toxicity [76], have obesogenic effects in stem cells [80], and induce oxidative stress and damage in several human cell types [77], among many other effects. Growing scientific evidence indicates that many of these BPA alternatives may be EDCs and ‘regrettable substitutions,’ being worse than the original parent compound and lacking proper testing, oversight, and regulation [81].

Few studies have examined these BPA alternatives and their effects on adipogenesis. Adipose-derived stem cells (ASCs; from fat tissue), a type of mesenchymal stem cell (MSC; from bone marrow, cord blood, connective tissue), are multipotent adult stem cells that have the ability to differentiate into various functional cell types [82]. BPS and BPAF have been reported to induce lipid accumulation similar to BPA [80], and affect the gene expression of murine and primary human ASCs and preadipocytes [83]. However, no studies have examined the effects of the newer BPA analogs including TMBPF on adipogenesis, and in a human stem cell model. Further, very limited non-industry-sponsored *in vitro* studies have been performed on TMBPF. Thus, we aimed to investigate the effects of TMBPF, BPAF, and BPA on adipogenesis in human stem cells to examine how exposure to these plastic and food-contact chemicals might be linked with the increasing fat gain in humans. We examined the effects of environmentally-relevant doses of BPA, BPAF, TMBPF, and 17β-estradiol, the most common natural estrogen, on adipogenesis in human female ASCs. Human ASCs (hASCs) can differentiate into adipocytes, among other cell types, thus making them an excellent model to study adipogenesis and fat cell development and growth. Here, we exposed hASCs to various low doses of these BPA analogs or 17β-estradiol, during adipocyte differentiation with chemically defined media. We then quantified adipogenesis and lipid production, as well as cell viability and apoptosis, and compared the effects and potencies of these bisphenols.

## 2. Results

### 2.1. Cell Viability with Low-Dose BPA, BPAF and TMBPF Exposure

**Figure 2:**
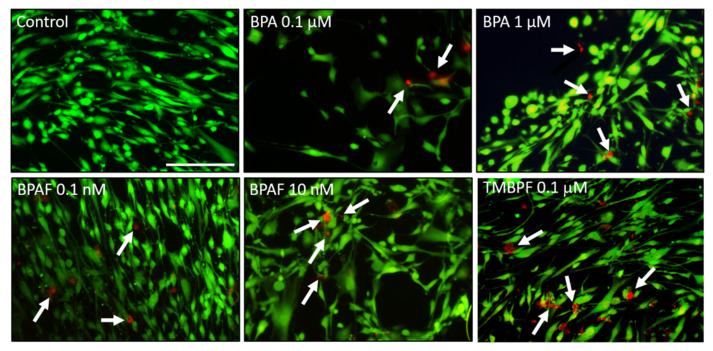
Cytotoxicity assay of BPA and BPA alternatives. Representative fluorescence images of hASCs treated with BPA at 0.1 µM and 1 µM, BPAF at 0.1 nM and 10 nM, or with TMBPF at 0.1 µM, after 24 h of exposure. Green indicates live cells and red indicates dead cells. Some cell death (see arrows) was found at each dose, especially with 0.1 μM TMBPF, but in general cells remained viable after 24 h of exposure to low-dose BPA and BPAF (200× magnification; scale bar = 150 μM; *n* = 3–4 slides/treatment; 3 trials).

As described in the methods, due to differences in potency, effective doses, and LC_50_s, different concentrations of the BPA analogs were used here. In order to determine the potential toxicity of low-dose BPA, BPAF, and TMBPF (subnanomolar to submicromolar), hASCs were exposed to various doses of the BPA analogs for 2 to 24 h. Following exposure, the cells were stained with the Live-Dead Cytotoxicity assay and imaged in order to quantify the percentage of live viable cells that remained. An increase in cell death and a decrease in cell viability were seen at higher doses of BPA (≥1 μM) and BPAF (≥1 nM) after 24 h (Figure 2). TMBPF at 0.1 µM showed some increases in cell death following 20 min, 2 h, and 24 h of exposure, but not complete cell death (Figure 2), whereas all higher doses resulted in significant toxicity and cell death (data not shown). Due to normal variation in cell attachment and variability in cell death with BPA and analog treatments, some wells/images appear to have lesser numbers of cells, as seen in Figure 2. An in-depth analysis of the cytotoxicity and apoptosis effects of these compounds can be found in our other recent work [71]. Here, in general, 80% or more of the low-dose BPA- and BPAF-treated cells and ~50–70% of the TMBPF-treated cells appeared to remain attached, growing, and viable following exposure. At the lowest tested doses (0.1 μM BPA, 0.1 nM BPAF, and 0.01 μM TMBPF) there was not a consistent significant decrease in cell viability compared to the ethanol control media-treated cells. From these experiments we determined that these low sublethal doses of BPA, BPAF, and TMBPF would be appropriate for use in differentiation studies, as they would not cause complete loss in cell viability.

### 2.2. Differentiation of Human ASCs into Adipocytes

**Figure 3:**
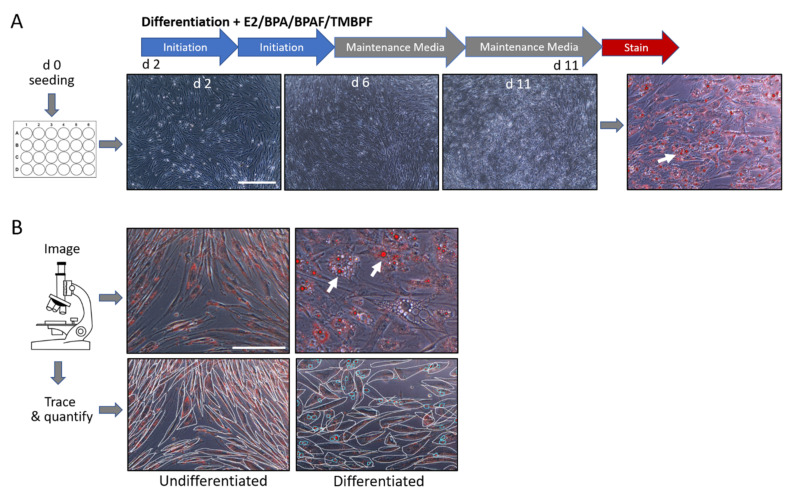
Timeline and quantification of the differentiation process of human ASCs into adipocytes. (**A**) Brightfield images of stem cells treated with cycles of initiation media and maintenance media. All cells were fixed, stained with Oil Red-O, imaged, and quantified for lipid vacuoles (white arrows). (**B**) Representative images of undifferentiated (**left**) and differentiated (**right**) cells after staining with Oil Red-O. Tracing of cells and lipid vacuoles in undifferentiated (**bottom left**) and differentiated (**bottom right**) cell populations allowed quantification of adipogenesis. Clear morphology changes occur in the differentiated cells compared to undifferentiated controls, as indicated by the round cell shape and lipid vacuoles (arrows in top image and circles in bottom image; 200× magnification; scale bar = 150 μM).

**Figure 4:**
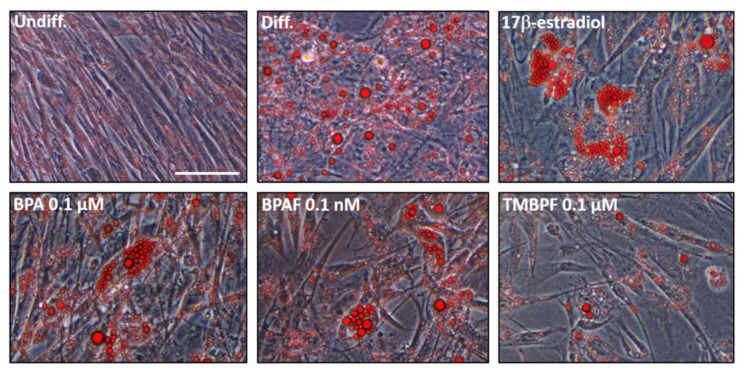
BPA and BPAF increase adipogenesis. Brightfield images of undifferentiated cells, control differentiated cells, and cells treated with either 10 µM 17β-estradiol, 0.1 µM BPA, 0.1 nM BPAF, or 0.1 µM TMBPF during the adipocyte differentiation process. Notice the relatively higher levels of adipogenesis and lipid production in the BPA- and BPAF-differentiated cells, similar to that of 17β-estradiol (see red spheres indicating large lipid vacuoles; 400× magnification; scale bar = 75 μM).

We first performed adipocyte differentiation using chemically defined medium and compared adipogenesis and lipid vacuole production among differentiated and control undifferentiated cells (Figure 3). A comparison of the control differentiated vs. undifferentiated cells showed that the cells were successfully undergoing differentiation into adipocytes. As depicted in Figure 3B, changes in cell morphology, as well as the presence of lipid droplets or vacuoles, exemplified the differences between the two controls. While the control undifferentiated cells maintained their long and thin morphology, the differentiated cells began to round-up during the differentiation process, and then accumulated many lipid vacuoles (Figure 3B; see red spheres). Because the cells were not differentiated for the full 21 days, not all of the control differentiated cells were observed to be mature adipocytes. However, it was clear that differentiation was successfully underway and almost three quarters of the cells had characteristic adipocyte morphology and had accumulated many lipid vacuoles (Figure 3B and Figure 4). To examine the effect of BPA, BPAF, and TMBPF on adipogenesis in human ASCs, cells were differentiated in the presence of these chemicals before being stained and analyzed as shown in Figure 4 (see also Supplementary Figures S1 and S2).

### 2.3. Low-Dose BPA and BPAF Increase Adipogenesis

**Figure 5:**
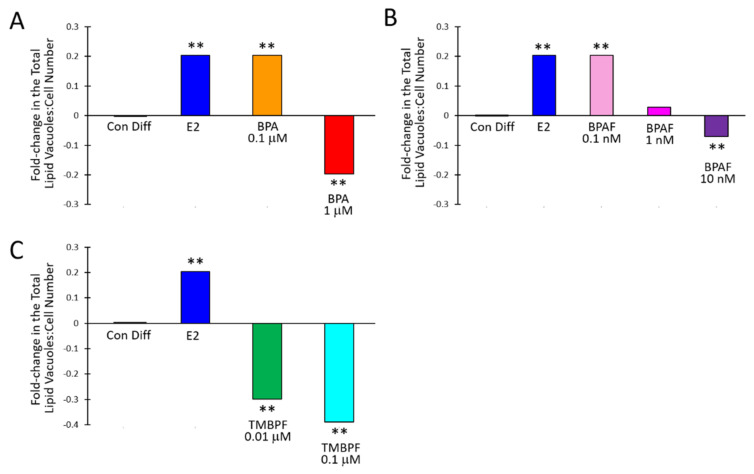
BPA and alternatives alter adipogenesis. The fold-change in the total ratio of lipid vacuoles to cell number in comparison to control differentiated cells (set to 0) after 11 days of differentiation and exposure to increasing doses of (**A**) BPA, (**B**) BPAF, or (**C**) TMBPF (*n* = 47–103 images/treatment; 4–10 wells/treatment; 2–3 trials; ** *p <* 0.005).

**Figure 6:**
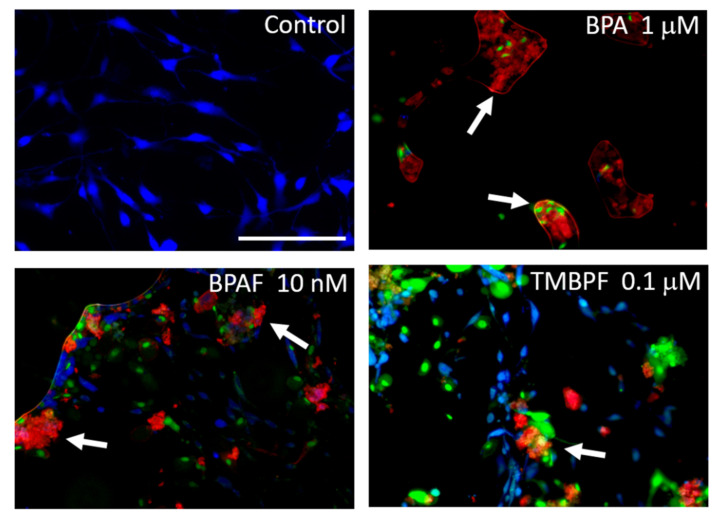
BPA and alternatives increase apoptosis. Representative fluorescent images of control, BPA-, BPAF-, and TMBPF-treated cells examined with an apoptosis-necrosis assay. Some cells treated with BPA, BPAF, and TMBPF exhibit clear signs of apoptosis and necrosis (see arrows; red = Apopxin Deep Red, indicates apoptosis; green = DNA Nuclear Green DCS1, indicates late-stage apoptosis and necrosis; blue = CytoCalcein Violet 450, indicates normal live cells) (200× magnification; scale bar = 150 μM).

We performed careful assessments of cell confluence and cell growth under brightfield microscopy, on each day of the differentiation protocol, as shown in Supplementary Figures S1 and S2. As observed from the brightfield images there were a great amount of cells attached and growing throughout the differentiation experiments, for all treatments. E2, BPA and BPAF significantly affected adipogenesis compared to the controls (Figure 4). The mean ratio of lipid vacuoles to cell number for the control undifferentiated cells was very low at 0.0015 ± 0.0057, whereas for the control differentiated cells, it was 0.74 ± 0.29 (*p <* 0.005). When comparing the total lipid vacuole number to total cell number across all trials, control undifferentiated cells were 0.0014, while the control differentiated cells were 0.78. E2 at 10 µM caused significantly increased adipogenesis compared to the differentiated controls with a mean ratio of lipid vacuoles to cell number of 0.92 ± 0.32 (*p* = 6.6 × 10^−5^) (Figure 5). Low concentrations of BPA and BPAF showed very similar results compared to E2. Visually, a greater number of lipid vacuoles was seen in the low-dose BPA- and BPAF-treated cells based on Oil Red-O staining (Figure 4). However, quantitative analyses revealed that the different doses of BPA had different effects on lipid accumulation (Figure 5A). BPA at 0.1 µM significantly increased adipogenesis with a mean ratio of lipid vacuoles to cell number of 0.91 ± 0.36 and a ratio of total lipid vacuole number to total cell number across trials of 0.97 (*p* = 0.001). This resulted in a 0.2-fold or 20% increase in adipogenesis (Figure 5A). On the other hand, 1 µM BPA significantly decreased adipogenesis, with a mean ratio of lipid vacuoles to cell number of 0.57 ± 0.30 and a ratio of total lipid vacuole number to total cell number of 0.63, which was a 20% reduction (*p* = 0.0008; Figure 5A). Higher doses of BPA may have more toxic effects compared to lower doses, thus contributing to an overall lower level of adipogenesis and lipid production. This was also confirmed with the apoptosis-necrosis assay, as 1 µM BPA treatment resulted in some apoptosis and necrosis as observed by the red and green cellular fluorescence after one hour of exposure (Figure 6). BPAF at 0.1 nM, led to a statistically significant 20% increase in adipogenesis (Figure 5B). At this dose, BPAF significantly increased lipid accumulation with a mean ratio of lipid vacuoles to cell number of 0.88 ± 0.31, and a ratio of total lipid vacuoles to total cell number of 0.94 (*p* = 0.002). However, BPAF at 1 nM, did not result in a significant increase in adipogenesis and did not differ significantly from control differentiated cells, while 10 nM BPAF led to a significant 7% reduction in adipogenesis (Figure 5B). Several doses of BPAF also led to an increase in the numbers of cells undergoing cell death and apoptosis as shown in Figure 6 (see red/pink and green cells).

### 2.4. TMBPF Decreases Adipogenesis and Shows Cytotoxicity in Stem Cells

Unlike BPA and BPAF, TMBPF significantly decreased adipogenesis and lipid accumulation at all concentrations tested. Based on staining and visual analysis, fewer lipid vacuoles accumulated in the TMBPF-treated cells, and cells generally lacked the rounded adipocyte morphology (Figure 4 and Supplementary Figure S2). Further, they appeared to be progressively dying throughout the differentiation process, as evidenced by the reduction in overall cell number and cell confluence (Figure 4 and Supplementary Figure S2). As observed in the brightfield images, we did find a general decline in the numbers of cells with TMBPF treatment throughout differentiation, but still sufficient numbers of live, attached cells per well (>50% confluence). Upon lipid vacuole quantification, 0.01 and 0.1 µM TMBPF both significantly decreased adipogenesis compared to controls by 30% and 40%, respectively (*p* = 0.0003 and 1.1 × 10^−9^, respectively) (Figure 5C). TMBPF at 0.01 µM had a mean ratio of lipid vacuoles to cell number of 0.56 ± 0.27 (total lipid vacuole number to cell number of 0.55); and at 0.1 µM had a mean ratio of lipid vacuoles to cell number of 0.47 ± 0.15 (total lipid vacuole number to cell number of 0.48, and a 40% reduction in adipogenesis) (Figure 5C). Throughout the differentiation process, there was decreased cell confluence and more cell death with TMBPF compared to the other bisphenols and controls. The apoptosis-necrosis assay confirmed that in as little as 1 h of exposure, some cells treated with 0.1 µM TMBPF exhibited clear signs of apoptosis (Figure 6; see red/pink and green cells). TMBPF appeared to have anti-adipogenic and cytotoxic effects on stem cells, thus causing overall reduced lipid production and greater levels of apoptosis.

## 3. Discussion

Here, we examined the effects of BPA and analogs on adipogenesis in human female adipose-derived stem cells and found that these chemicals significantly impacted fat cell development and lipid accumulation. Our work is the first to examine the effects of TMBPF on adipogenesis. We found that low-dose BPA and BPAF had obesogenic effects and significantly increased adipogenesis and lipid vacuole production. Conversely, TMBPF and higher doses of BPA and BPAF significantly decreased adipogenesis and lipid vacuole production. Importantly, low-dose BPA (0.1 μM) and BPAF (0.1 nM) showed very similar effects as 17β-estradiol, the most common natural estrogen, providing further evidence for their estrogen-mimicking, endocrine-disrupting effects in human stem cells.

This data is consistent with previous studies reporting a link between exposure to low-dose BPA and obesity, BPA-induced enhancement of preadipocyte differentiation, and the expression of adipogenesis-associated genes and transcription factors [32,62,63]. BPA promotes adipogenesis in 3T3-L1 stem cells by glucocorticoid-receptor activation, a process that is central to adipocyte differentiation [84]. When these cells were cultured in the presence of 1 nM BPA for 21 days before differentiation and during the differentiation process, there was increased expression of adipogenic genes. The resulting adipocytes also had impaired metabolic functioning and increased pro-inflammatory cytokine expression [62]. There was also a greater expression of adipogenic transcription factors. Stem cells differentiated into adipocytes in the presence of BPA also resulted in more significant lipid accumulation [63].

Additionally, BPA interferes with normal insulin action in differentiated rat and human adipocytes [85]. Ohlstein et al. found that BPA at 1 μM increases adipogenesis in human ASCs and likely acts through an estrogen-mediated pathway, affecting the expression of adipogenesis-associated genes including insulin-like growth factor 1 (IGF1) and others, as well as increases the expression of lipoprotein lipase [32]. Although we found a significant 20% increase in adipogenesis with 0.1 μM BPA, unlike Ohlstein et al., we found a decrease in adipogenesis at 1 μM BPA. Thus, human ASCs may be more sensitive to BPA and analogs than many other cell types. Further, BPA may have more toxic effects on hASCs at this concentration, which overshadows the endocrine-disrupting effects at higher doses. Indeed, we found significant cell death and apoptosis at 1 μM BPA. Notably, this is in line with our other recent work in adult rat and human stem cells [71].

The doses of BPA and BPAF tested here are lower than those of previous studies, and are comparable to the levels detected in our surrounding environment and in human fluids, highlighting the physiological relevance of our findings [18,25,48,86,87]. Concentrations of BPA in aquatic systems and water samples have been found in the high nM to low µM (ppb) range, from 1 to 21 µg/L (4–90 nM) [18]. BPAF has been detected at much lower levels, generally in the low pM to nM (ppt) range, from 1 to 246 ng/L (0.003–0.73 nM) [86]. To date, no studies have explored the levels of TMBPF in the environment or human fluids, likely because of its recent use. It is of note that in the relatively higher doses of BPA and BPAF and all doses of TMBPF tested, there was a correlation between decreased adipogenesis and increased apoptosis, indicating some likely toxicity even at these relatively low, environmentally-relevant doses.Previous studies have examined the endocrine-disrupting actions of BPS and BPAF and found they increase adipogenesis and disrupt the metabolic functioning of both mature adipocytes and preadipocytes [80,83,88,89]. BPAF disrupts lipid and carbohydrate metabolism in adipocytes and activates inflammatory signaling pathways that degrade metabolic activity in human fat cells. BPAF also increases the expression of critical adipogenic markers in murine preadipocytes [88,89]. While our study did not examine these signaling pathways, our results are consistent with these findings. They exemplify BPAF’s role as an EDC by increasing lipid accumulation, very similar to that of 17β-estradiol. Notably, we found that BPAF and E2 both increased adipogenesis by 20%. For BPAF, this effective dose was 1,000-fold lower than that of BPA, highlighting its increased potency. These specific trends among bisphenol potencies and toxicities are consistent among ours and other’s findings when exploring the effects of the compounds on adipogenesis, embryo development and cell division, and cytotoxicity and apoptosis in human stem cells [63,68,69,70,71,80,88,89].

Corporations propose TMBPF is a safe, low-toxicity replacement for BPA. It is one of the newest BPA alternatives currently being used in metal food-contact coatings and other products [64,90]. It is already estimated to be in approximately 5% of beverage and food cans worldwide [90]. However, few independent investigations have examined its effects on human cells, and none have examined its effects on adipogenesis. Some conflicting findings have reported that TMBPF lacks estrogenic and anti-androgenic activity associated with other bisphenols, both in vitro and in vivo [65,66,91]. Dietary toxicity studies for 90 days in 8-week-old rats reported no systemic toxicity or significant alterations to endocrine endpoints [66]. However, TMBPF significantly increased liver and kidney weights at the end of the study in animals treated with 1,000 mg/kg BW/day that persisted in males at the end of the 28-day recovery period. It also led to dose-dependent increases in thymus cell proliferation and ovarian follicular cysts, which appeared to subside after the recovery period [66]. In a study by Soto et al., TMBPF did not show estrogen-agonist or -antagonist activity in an estrogen receptor-transactivation assay, nor did it cause changes in puberty or mammary gland development in male and female rats [65].

In contrast, Szafran et al., using several human cell-based, high-throughput systems, found that TMBPF had both anti-estrogenic and anti-androgenic activity in HeLa, breast cancer (MCF7), and prostate cancer (LNCaP) cell lines, respectively [91]. In addition, they reported significant cell loss/death in prostate cancer cells with 5 μM TMBPF, but minimal cell loss at 2 μM and lower. Here, we found that TMBPF differed in its effects on fat cell development from 17β-estradiol and the other bisphenols. While E2, BPA, and BPAF increased adipogenesis, all doses of TMBPF tested (0.01–1 μM) had anti-adipogenic effects, causing a significant 30–40% decrease in adipogenesis and lipid production.

TMBPF’s distinct effect on adipogenesis likely indicates a different chemical and toxicological profile compared to other bisphenols and we hypothesize it is acting through non-estrogen-mediated pathways. Indeed, our other recent work found that TMBPF was 100-fold more cytotoxic and potent than BPA in human stem cells and activated apoptosis via caspase-6-mediated, non-estrogenic pathways [71]. Similarly, continuous exposure to TMBPF during differentiation resulted in increasing cell death rates, measured by qualitative assessments of cell confluence and quantitative assessments of cell viability and apoptosis. Mortality at least partially explains the decreased levels of adipogenesis and highlights TMBPF’s significantly higher potency than BPA. We normalized the overall proportion of differentiating adipocytes by factoring in the ratio of lipid vacuoles to total numbers of cells to help account for cell loss/reduction in viable cells from well to well. For example, a well with 1000 cells and 1000 lipid vacuoles would have a ratio of 1, whereas even if a well had significant cell loss and only 100 cells with a similar proportion of lipid vacuoles of 100, the well would similarly have a ratio of 1. The reduced ratios therefore, indicate that TMBPF’s effects are not only via cytotoxicity. While TMBPF indeed seems to lack the estrogen-like activity of many other bisphenols, it is unclear whether this compound completely lacks other endocrine-disrupting actions as well. However, TMBPF clearly has anti-adipogenic, cytotoxic, and apoptosis-inducing effects on human stem cells at very low doses, warranting further toxicological characterization. Future investigations will further examine TMBPF’s underlying signaling pathways and mechanisms of action.

At the cellular level, BPA and some of its alternatives may act as environmental toxins and obesogens. BPA, BPAF, and TMBPF all altered the ability of human stem cells to differentiate into fat cells and produce lipids. In addition, BPA and BPAF had obesogenic and EDC effects similar to that of E2, while TMBPF decreased fat cell development. These findings provide one potential mechanistic explanation for the connection between bisphenol exposure and fat gain in humans. Given the widespread presence of BPA and BPA replacements in plastic and consumer products, the human body, and the environment, as well as the tendency for these chemicals to accumulate in fat cells and tissues, these results have direct implications for better understanding the etiologies and correlates of human obesity. It is vital that we continue to expand our knowledge on these commonly used BPA replacements and their effects on humans, animals, and the environment. As it has become one of the top human health crises, we must continue to explore the links between obesity and chemical exposures. This work underscores the need for greater regulation of compounds and their analogs rather than on individual chemicals. Further toxicological studies, better guidelines for non-consumable products, and greater public awareness are necessary to develop safer chemicals and everyday products.

## 4. Materials and Methods

### 4.1. Preparation of Chemicals and Reagents

Stock solutions, at 10 mM, of BPA (133027; >97% purity), BPAF (90477; >99% purity), TMBPF (M1099; >98% purity), and 17β-estradiol (E8875; >98% purity) were prepared in 95% ethanol in glass bottles. The stock solutions were then diluted with cell culture and differentiation media to the desired treatment concentrations for the differentiation studies, and were prepared fresh on the day of the experiments. Ethanol at 0.01% was added to the control differentiated and undifferentiated solutions for each experiment to ensure that all control and treated cells were exposed to the same concentration of ethanol. Chemicals were purchased from Sigma Aldrich (St. Louis, MO, USA; BPAF, BPA, and 17β-estradiol) and Tokyo Chemical Industry (Tokyo, Japan; TMBPF).

### 4.2. Stem Cell Isolation and Cell Culture

Human adipose-derived stem cells (hASCs) were purchased from Lifeline Cell Technology (FC-0062; Frederick, MD, USA). These cells were isolated from mature adipocytes through liposuction surgery from two consented adult females and then dedifferentiated back into stem cells. The cells were maintained with aseptic cell culture, and grown in culture medium containing basal medium, FGF, insulin, ascorbic acid, L-glutamine, hydrocortisone hemisuccinate, FBS, and an antimicrobial supplement (LL-0011; Lifeline Cell Technology). Cells were split enzymatically and passaged using 0.05% trypsin/0.02% EDTA (CM-0017; Lifeline Cell Technology) and trypsin neutralizing solution (CM-0018; Lifeline Cell Technology) on a weekly basis and were maintained at a confluency of 70–80% for ideal growth conditions. Cells were cultured at 37 °C in an incubator with 5% CO_2_.

Rat adipose-derived stem cells (rASCs) were obtained and isolated from the inguinal fat region of female Lewis rats by surgery and enzymatic digestion, as previously described [82] (kindly provided by Dr. David Sahar, U.C. Davis Medical Center, CA, USA). All animal experimental procedures were approved and performed in accordance with the U.C. Davis School of Medicine Institutional Animal Care and Use Committee (IACUC). Rat ASCs from passages II–IV were used for all subsequent experiments. Cells were seeded at a density of 2.5 × 10^5^ cells/cm^2^ and cultured in growth medium consisting of α-modified minimal essential medium (α-MEM) (SH20626.01; GE Healthcare Life Sciences; Marlborough, MA, USA) with 10% fetal bovine serum (FBS) (10437-010; Gibco/Life Technologies Corporation; Waltham, MA, USA) and 100X Penicillin-Streptomycin-Amphotericin B (pen/strep/amp) (10378-016; Gibco/Life Technologies Corporation). Upon reaching 90% confluence, the cells were passaged, by washing with phosphate-buffered saline (PBS) (21-040-CM; Corning; Corning, NY, USA) and dissociated by incubation with 0.5% Trypsin-EDTA (T3924; Sigma-Aldrich; St. Louis, MO, USA) for 5 min at 37 °C with 5% CO_2_. Following trypsinization, trypsin was neutralized with growth medium, and cells were transferred to a 15 mL conical tube and centrifuged for five minutes at 1200 rpm. The supernatant was aspirated off and the pellet was resuspended in growth medium. The cells were then seeded on new plates, grown in a 37 °C incubator with 5% CO_2_, and the media was changed every two days.

### 4.3. BPA, BPAF and TMBPF Cytotoxicity

Preliminary rangefinder assays were conducted with each chemical to find the range of concentrations over which sublethal cellular effects and adipogenesis might occur. Our previous studies on embryo cell cleavage division and development and rat and human adult ASCs also provided initial ranges to test in these preliminary studies [69,70,71]. Due to differences in potency, toxicity, effective doses, and LC_50_s, different concentrations of the BPA analogs were used here. Using the same concentrations of all chemicals, while the simplest experimental design, would have resulted in massive cell death for compounds such as BPAF, obscuring any effects on adipogenesis. Therefore, in the final experiments, not all chemicals were used at the same concentrations. Further, to aid in these rangefinder assays, preliminary cytotoxicity tests were performed with BPA, BPAF, and TMBPF using the Live/Dead Viability-Cytotoxicity Kit for mammalian cells (L3224; Thermo Fisher Scientific; Waltham, MA, USA). Human ASCs were plated in 6-well plates on glass coverslips (22 × 22 mm, size 1.5) and grown until they reached 70–80% confluence. The cells were incubated with BPA (0.1 and 1 μM), BPAF (0.1, 1 and 10 nM), or TMBPF (0.1 and 1 μM), for 2 to 24 h. After chemical exposure, the cells were washed with PBS and treated with 2 μM calcein AM and 1 μM ethidium homodimer-1 in PBS for 30 min, protected from light. The live and dead cells were counted based on the presence of green or red fluorescence due to the calcein AM and ethidium homodimer-1 dye, respectively, and the percentage of live viable cells across treatments and replicates was quantified. Apoptosis was further determined using the Apoptosis-Necrosis Assay Kit for mammalian cells (ab176749; Abcam; Burlingame, CA, USA). After initial exposure to BPA analogs for 60 min, and following the manufacturer’s instructions, the cells were washed with the assay buffer and incubated with a master mix of Apopxin Deep Red Indicator (from 100× stock), Nuclear Green DCS1 (from 200× stock), and CytoCalcein Violet 450 (from 200× stock). The stock of CytoCalcein Violet 450 was prepared using dimethyl sulfoxide (DMSO; D2650, Sigma-Aldrich). The cells were incubated at room temperature protected from light for 60 min, and then mounted on slides and imaged immediately using fluorescent microscopy. Cells were analyzed using this tri-color assay for the clear signs of apoptosis and necrosis. Red cells expressing Apopxin Deep Red indicated apoptosis, green cells expressing DNA Nuclear Green DCS1 indicated late-stage apoptosis and necrosis, and blue cells expressing CytoCalcein Violet 450, indicated normal cell viability.

### 4.4. Adipocyte Differentiation of Rat ASCs

As shown in Supplementary Figure S3, to examine the multipotential differentiation of ASCs, and the proof of principle and methodology of differentiation into adipocytes in a more cost-effective system, we first used the rat ASC model and a previously published protocol, before beginning studies in more costly human stem cells [82]. Female rat ASCs were seeded in a 6-well plate at a density of 2 × 10^5^ cells/cm^2^, with fresh α-MEM growth medium exchanged every two to three days. Mesenchymal Stem Cell Adipogenic Differentiation Basal Medium A (GUXMX-03031; Cyagen Biosciences Inc.; Santa Clara, CA, USA) was used to induce adipogenic differentiation after the cells reached 100% confluence. After three days, the induction media was aspirated off and replaced with Mesenchymal Stem Cell Adipogenic Differentiation Basal Medium B maintenance media (GUXMX-03032; Cyagen Biosciences Inc; Santa Clara, CA, USA). The induction A/maintenance B media were alternated for three cycles of 3 days/1 day (Supplementary Figure S3). The differentiated rASCs were fixed with 4% paraformaldehyde (PFA) and stained with Oil Red-O working solution for 30 min (Cyagen Biosciences Inc.; 3:2 dilution with distilled water and filtered with filter paper), and then imaged (Supplementary Figure S3).

### 4.5. Adipocyte Differentiation of Human ASCs Exposed to BPA Analogs or 17β-Estradiol

All experiments were performed in 24-well plates. Human ASCs were thawed and seeded directly from cryovials to experimental well plates at a density of 20,000 cells per cm^2^ in normal cell culture media (day 0; Figure 3A). A protocol for adipogenesis was used, along with the Adipolife DfKt-1 Adipogenesis kit from Lifeline Cell Technology, following the manufacturer’s instructions (LL-0050; Lifeline Cell Technology). The differentiation protocol began 2 days after inoculation (d 2). As shown in Figure 3A, initiation media was used for the first 4 days of differentiation (d 2–6), and maintenance media was used for the rest of the differentiation process (d 6–11). Estradiol at 10 μM, a physiologically-relevant concentration known to induce adipogenesis, was used as a positive control. On day 2, normal cell culture media containing 0.01% ethanol was added to the control undifferentiated wells, and initiation differentiation media with 0.01% ethanol was added to the control differentiated wells. Additionally, on day 2, initiation differentiation media with estradiol was added to the positive control wells, and initiation differentiation media with increasing doses of BPA (0.1 and 1 μM), BPAF (0.1, 1 and 10 nM), or TMBPF (0.01 and 0.1 μM) was added to the treatment wells. Initiation media was used for 4 days and then was replaced by maintenance media containing E2 or the bisphenols for the next 5 days. The cell culture and differentiation media were replaced every 2 days for the first half of differentiation, and then every 3 days for the last half. The hASCs were differentiated for a total of 11 days before being washed with PBS and fixed with 4% PFA (Figure 3A). An 11-day protocol instead of 21 days (2 days of growth and 9 days of differentiation) was carried out as we wanted to carefully investigate any differences in differentiation speed and efficiency among the wells treated with various doses of the BPA alternatives. After the cells were fixed, they were stained with Oil Red-O (01391; Sigma Aldrich; St. Louis, MO, USA) for 40 min, and imaged (Figure 3A and Supplementary Figures S1 and S2).

### 4.6. Adipocyte and Lipid Quantification and Statistical Analysis

Following staining, the cells were imaged on a fluorescent EVOS M5000 inverted microscope with the addition of an RGB filter (Thermo Fisher Scientific, Waltham, MA, USA), in order to examine the number of adipocytes and their accumulation of lipid vacuoles. Ten to fifteen images were captured per well. The lipid vacuoles and total number of cells were quantified using Microsoft OneNote by the tracking of both lipid vacuoles and cells (Figure 3B). The ratio of lipid vacuoles to total cell number was calculated to compare the levels of adipogenesis and lipid accumulation in the control undifferentiated, control differentiated, E2-treated, and the BPA-, BPAF-, and TMBPF-treated cells. As we wanted to compared the effects of E2 and the bisphenols to the normally differentiated cells, we normalized the results against control differentiated cells by setting their ratio of total lipid vacuoles to total cell number across all trials to 0, and calculated the difference between each treated group and controls and their respective positive or negative fold-change. All treatments were performed in duplicate or triplicate for each trial. The Student’s t-test (two-tailed) was performed on all test groups versus the control, followed by a One-Way ANOVA and Tukey’s Multiple Comparison to determine statistically significant differences between treatments, using Microsoft Excel and the statistical program R. *p* values of less than 0.05 (*) and 0.005 (**) were considered statistically significant. All results were expressed as the fold-change in the total ratio of lipid vacuoles to cell number, and the mean ± standard deviation of the mean for at least 2–3 independent trials.

## Data Availability

All relevant summary data are within this article. Additional raw and representative data contributing to the summary figures are included in the Supplementary Figures.

## Supplementary Materials

The following are available online at https://www.mdpi.com/article/10.3390/ijms22105363/s1.

## References

[1] Vogel S.A. (2009). The politics of plastics: The making and unmaking of Bisphenol A “Safety”. Am. J. Public Health.

[2] Janesick A., Bloomberg B. (2012). Obesogens, stem cells, and the developmental programming of obesity. Int. J. Androl..

[3] Braun J.M., Hauser R. (2011). Bisphenol A and children’s health. Curr. Opin. Pediatr..

[4] Flint S., Markle T., Thompson S., Wallace E. (2012). Bisphenol A exposure, effects, and policy: A wildlife perspective. J. Environ. Manag..

[5] Onundi Y., Drake B.A., Malecky R.T., DeNardo M.A., Mills M.R., Kundu S., Ryabov A.D., Beach E.S., Horwitz C.P., Simonich M.T. (2017). A multidisciplinary investigation of the technical and environmental performances of TAML/peroxide elimination of Bisphenol A compounds from water. Green Chem..

[6] Chianese R., Troisi J., Richards S., Scafuro M., Fasano S., Guida M., Pierantoni R., Meccariello R. (2018). Bisphenol A in Reproduction: Epigenetic Effects. Curr. Med. Chem..

[7] Vandenberg L.N., Chahoud I., Heindel J.J., Padmanabhan V., Paumgartten F.J.R., Schoenfelder G. (2010). Urinary, Circulating, and Tissue Biomonitoring Studies Indicate Widespread Exposure to Bisphenol A. Environ. Health Perspect..

[8] Almeida S., Raposo A., Almeida-González M., Carrascosa C. (2018). Bisphenol A: Food Exposure and Impact on Human Health. Compr. Rev. Food Sci. Food Saf..

[9] Rubin B.S., Schaeberle C.M., Soto A.M. (2019). The Case for BPA as an Obesogen: Contributors to the Controversy. Front. Endocrinol..

[10] Cao X.L., Perez-Locas C., Dufresne G., Clement G., Popovic S., Beraldin F., Dabeka R.W., Feeley M. (2011). Concentrations of bisphenol A in the composite food samples from the 2008 Canadian total diet study in Quebec City and dietary intake estimates. Food Addit. Contam..

[11] Liao C., Kannan K. (2013). Concentrations and Profiles of Bisphenol A and Other Bisphenol Analogues in Foodstuffs from the United States and Their Implications for Human Exposure. J. Agric. Food Chem..

[12] Geens T., Aerts D., Berthot C., Bourguignon J.-P., Goeyens L., Lecomte P., Maghuin-Rogister G., Pironnet A.-M., Pussemier L., Scippo M.-L. (2012). A review of dietary and non-dietary exposure to bisphenol-A. Food Chem. Toxicol..

[13] Hanaoka T., Kawamura N., Hara K., Tsugane S. (2002). Urinary bisphenol A and plasma hormone concentrations in male workers exposed to bisphenol A diglycidyl ether and mixed organic solvents. Occup. Environ. Med..

[14] Ozaki A., Yamaguchi Y., Fujita T., Kuroda K., Endo G. (2004). Chemical analysis and genotoxicological safety assessment of paper and paperboard used for food packaging. Food Chem. Toxicol..

[15] Corrales J., Kristofco L.A., Steele W.B., Yates B.S., Breed C.S., Williams E.S., Brooks B.W. (2015). Global assessment of bisphenol A in the environment: Review and analysis of its occurrence and bioaccumulation. Dose Response.

[16] Liao C., Liu F., Moon H.-B., Yamashita N., Yun S., Kannan K. (2012). Bisphenol Analogues in Sediments from Industrialized Areas in the United States, Japan, and Korea: Spatial and Temporal Distributions. Environ. Sci. Technol..

[17] Jin H., Zhu L. (2016). Occurrence and partitioning of bisphenol analogues in water and sediment from Liaohe River Basin and Taihu Lake, China. Water Res..

[18] Fromme H., Küchler T., Otto T., Pilz K., Müller J., Wenzel A. (2002). Occurrence of phthalates and bisphenol A and F in the environment. Water Res..

[19] Lee H.-B., Peart T.E. (2000). Bisphenol A Contamination in Canadian Municipal and Industrial Wastewater and Sludge Samples. Water Qual. Res. J..

[20] Vandenberg L.N., Hauser R., Marcus M., Olea N., Welshons W.V. (2007). Human exposure to bisphenol A (BPA). Reprod. Toxicol..

[21] Yamazaki E., Yamashita N., Taniyasu S., Lam J., Lam P.K., Moon H.-B., Jeong Y., Kannan P., Achyuthan H., Munuswamy N. (2015). Bisphenol A and other bisphenol analogues including BPS and BPF in surface water samples from Japan, China, Korea and India. Ecotoxicol. Environ. Saf..

[22] Mathieu-Denoncourt J., Wallace S.J., De Solla S.R., Langlois V.S. (2016). Influence of Lipophilicity on the Toxicity of Bisphenol A and Phthalates to Aquatic Organisms. Bull. Environ. Contam. Toxicol..

[23] Bernier M.R., Vandenberg L.N. (2017). Handling of thermal paper: Implications for dermal exposure to bisphenol A and its alternatives. PLoS ONE.

[24] Toner F., Allan G., Dimond S.S., Waechter J.M., Beyer D. (2018). In Vitro percutaneous absorption and metabolism of Bisphenol A (BPA) through fresh human skin. Toxicol. In Vitro.

[25] Zhang Z., Alomirah H., Cho H.-S., Li Y.-F., Liao C., Minh T.B., Mohd M.A., Nakata H., Ren N., Kannan K. (2011). Urinary Bisphenol A Concentrations and Their Implications for Human Exposure in Several Asian Countries. Environ. Sci. Technol..

[26] Xue J., Wu Q., Sakthivel S., Pavithran P.V., Vasukutty J.R., Kannan K. (2015). Urinary levels of endocrine-disrupting chemicals, including bisphenols, bisphenol A diglycidyl ethers, benzophenones, parabens, and triclosan in obese and non-obese Indian children. Environ. Res..

[27] Fisher M., Arbuckle T.E., MacPherson S., Braun J.M., Feeley M., Gaudreau É. (2019). Phthalate and BPA Exposure in Women and Newborns through Personal Care Product Use and Food Packaging. Environ. Sci. Technol..

[28] Johns L.E., Ferguson K.K., Meeker J.D. (2016). Relationships Between Urinary Phthalate Metabolite and Bisphenol A Concentrations and Vitamin D Levels in U.S. Adults: National Health and Nutrition Examination Survey (NHANES), 2005–2010. J. Clin. Endocrinol. Metab..

[29] Koch H.M., Kolossa-Gehring M., Schröter-Kermani C., Angerer J., Brüning T. (2012). Bisphenol A in 24 h urine and plasma samples of the German Environmental Specimen Bank from 1995 to 2009: A retrospective exposure evaluation. J. Expo. Sci. Environ. Epidemiol..

[30] Longnecker M., Harbak K., Kissling G., Hoppin J., Eggesbo M., Jusko T., Eide J., Koch H. (2013). The concentration of bisphenol A in urine is affected by specimen collection, a preservative, and handling. Environ. Res..

[31] Lehmler H.J., Liu B., Gadogbe M., Bao W. (2018). Exposure to bisphenol A, bisphenol F, and bisphenol S in U. S. adults and children: The national health and nutrition examination survey 2013–2014. ACS Omega.

[32] Ohlstein J.F., Strong A.L., McLachlan J.A., Gimble J.M., Burow M.E., Bunnell B.A. (2014). Bisphenol A enhances adipogenic differentiation of human adipose stromal/stem cells. J. Mol. Endocrinol..

[33] Gould J.C., Leonard L.S., Maness S.C., Wagner B.L., Conner K., Zacharewski T., Safe S., McDonell D.P., Gaido K.W. (1998). Bisphenol A interacts with the estrogen receptor alpha in a distinct manner from estradiol. Mol. Cell. Endocrinol..

[34] Pouzand F., Thierry-Mieg M., Burga K., Vérines-Jouin L., Fiore K., Beausoleil C., Michel C., Rousselle C., Pasquier E. (2018). Concerns related to ED-Mediated effects of Bisphenol A and their regulatory consideration. Mol. Cell. Endocrinol..

[35] Rubin B.S. (2011). Bisphenol A: An endocrine disruptor with widespread exposure and multiple effects. J. Steroid Biochem. Mol. Biol..

[36] Karwacka A., Zamkowksa D., Radwan M., Jurewicz J. (2019). Exposure to modern, widespread environmental endocrine disrupting chemicals and their effect on the reproductive potential of women: An overview of current epidemiological research. Hum. Fertil..

[37] Moklin E., Ehrlich S., Williams P.L., Petrozza J.C., Wright D.L., Calafat A.M., Ye X., Hauser R. (2010). Urinary bisphenol A concentrations and ovarian response among women undergoing IVF. Int. J. Androl..

[38] Mendiola J., Jørgensen N., Andersson A.-M., Calafat A.M., Ye X., Redmon J.B., Drobnis E.Z., Wang C., Sparks A., Thurston S.W. (2010). Are Environmental Levels of Bisphenol A Associated with Reproductive Function in Fertile Men?. Environ. Health Perspect..

[39] Zbucka-Kretowska M., Zbucki R., Parfieniuk E., Maslyk M., Lazarek U., Miltyk W., Czerniecki J., Wolczynski S., Kretowski A., Ciborowski M. (2018). Evaluation of Bisphenol A influence on endocannabinoid system in pregnant women. Chemosphere.

[40] Kitamura S., Suzuki T., Sanoh S., Kohta R., Jinno N., Sugihara K., Yoshihara S., Fujimoto N., Watanabe H., Ohta S. (2005). Comparative Study of the Endocrine-Disrupting Activity of Bisphenol A and 19 Related Compounds. Toxicol. Sci..

[41] Kardas F., Bayram A.K., Demirci E., Akin L., Ozmen S., Kendirci M., Canpolat M., Oztop D.B., Narin F., Gumus H. (2016). Increased Serum Phthalates (MEHP, DEHP) and Bisphenol A Concentrations in Children With Autism Spectrum Disorder: The Role of Endocrine Disruptors in Autism Etiopathogenesis. J. Child Neurol..

[42] Thongkorn S., Kanlayaprasit S., Jindatip D., Tencomnao T., Hu V.W., Sarachana T. (2019). Sex Differences in the Effects of Prenatal Bisphenol A Exposure on Genes Associated with Autism Spectrum Disorder in the Hippocampus. Sci. Rep..

[43] Jenkins S., Raghuraman N., Eltoum I., Carpenter M., Russo J., Lamartiniere C.A. (2009). Oral Exposure to Bisphenol A Increases Dimethylbenzanthracene-Induced Mammary Cancer in Rats. Environ. Health Perspect..

[44] Shafei A., Ramzy M.M., Hegazy A.I., Husseny A.K., El-Hadary U.G., Taha M.M., Mosa A.A. (2018). The molecular mechanisms of action of the endocrine disrupting chemical bisphenol A in the development of cancer. Gene.

[45] Zhang K.S., Chen H.Q., Chen Y.S., Qiu K.F., Zheng X.B., Li G.C., Yang H.D., Wen C.J. (2014). Bisphenol A stimulates human lung cancer cell migration via upregulation of matrix metalloproteinases by GPER/EGFR/ERK1/2 signal pathway. Biomed. Pharmacother..

[46] Obesity and Overweight Factsheets. https://www.who.int/news-room/fact-sheets/detail/obesity-and-overweight.

[47] Jacobson M.H., Woodward M., Bao W., Liu B., Trasande L. (2019). Urinary Bisphenols and Obesity Prevalence among U.S. Children and Adolescents. J. Endocr. Soc..

[48] Calafat A., Ye X., Wong L.Y., Reidy J.A., Needham L.L. (2008). Exposure of the U.S. Population to bisphenol a and 4-Tert-Octylphenol: 2003–2004. Environ. Health Perspect..

[49] Kim K.Y., Lee E., Kim Y. (2019). The Association between Bisphenol A Exposure and Obesity in Children—A Systematic Review with Meta-Analysis. Int. J. Environ. Res. Public Health.

[50] Bhandari R., Xiao J., Shankar A. (2013). Urinary Bisphenol A and Obesity in US Children. Am. J. Epidemiol..

[51] Van Woerden I., Bruening M., Montresor-López J., Payne-Sturges D.C. (2019). Trends and disparities in urinary BPA concentrations among U.S. emerging adults. Environ. Res..

[52] Hagobian T., Smouse A., Streeter M., Wurst C., Schaffner A., Phelan S. (2017). Randomized Intervention Trial to Decrease Bisphenol A Urine Concentrations in Women: Pilot Study. J. Women Health.

[53] Do M.T., Chang V.C., Mendez M.A., De Groh M. (2017). Urinary bisphenol A and obesity in adults: Results from the Canadian Health Measures Survey. Health Promot. Chronic Dis. Prev. Can..

[54] Khan J., Salhotra S., Goswami P., Akhter J., Jahan S., Gupta S., Sharma S., Banerjee B.D., Parvez S., Gupta S. (2019). Bisphenol A triggers axonal injury and myelin degeneration with concomitant neurobehavioral toxicity in C57BL/6J male mic. Toxicology.

[55] Nishikawa M., Iwano H., Yanagisawa R., Koike N., Inoue H., Yokota H. (2010). Placental Transfer of Conjugated Bisphenol A and Subsequent Reactivation in the Rat Fetus. Environ. Health Perspect..

[56] Aris A. (2014). Estimation of bisphenol A (BPA) concentrations in pregnant women, fetuses and nonpregnant women in Eastern Townships of Canada. Reprod. Toxicol..

[57] Lee J., Choi K., Park J., Moon H.-B., Choi G., Lee J.J., Suh E., Kim H.-J., Eun S.-H., Kim G.-H. (2018). Bisphenol A distribution in serum, urine, placenta, breast milk, and umbilical cord serum in a birth panel of mother–neonate pairs. Sci. Total Environ..

[58] Bertoli S., Leone A., Battezzati A. (2015). Human Bisphenol A exposure and the “Diabetes Phenotype”. Dose Response.

[59] Fernandez M.F., Arrebola J., Taoufiki J., Navalón A., Ballesteros O., Pulgar R., Vilchez J., Olea N. (2007). Bisphenol-A and chlorinated derivatives in adipose tissue of women. Reprod. Toxicol..

[60] Jackson E., Shoemaker R., Larian N., Cassis L. (2017). Adipose Tissue as a Site of Toxin Accumulation. Compr. Physiol..

[61] Wang L., Asimakopoulos A.G., Kannan K. (2015). Accumulation of 19 environmental phenolic and xenobiotic heterocyclic aromatic compounds in human adipose tissue. Environ. Int..

[62] Ariemma F., D’Esposito V., Liguoro D., Oriente F., Cabaro S., Liotti A., Cimmino I., Longo M., Beguinot F., Formisano P. (2016). Low-Dose Bisphenol-A Impairs Adipogenesis and Generates Dysfunctional 3T3-L1 Adipocytes. PLoS ONE.

[63] Desai M., Ferrini M.G., Jellyman J.K., Han G., Ross M.G. (2018). In Vivo and In Vitro bisphenol A exposure effects on adiposity. J. Dev. Orig. Health Dis..

[64] ValPure V70 Series Next Generation Coatings. https://www.valsparpackaging.com/assets/files/valpure_v70_bulletin_2017_v4.pdf.

[65] Soto A.M., Schaeberle C., Maier M.S., Sonnenschein C., Maffini M.V. (2017). Evidence of Absence: Estrogenicity Assessment of a New Food-Contact Coating and the Bisphenol Used in Its Synthesis. Environ. Sci. Technol..

[66] Maffini M.V., Canatsey R.D. (2020). An expanded toxicological profile of tetramethyl bisphenol F (TMBPF), a precursor for a new food-contact metal packaging coating. Food Chem. Toxicol..

[67] Hong L., Wang Y., Wang L., Zhang H., Na H., Zhang Z. (2017). Synthesis and characterization of a novel resin monomer with low viscosity. J. Dent..

[68] Moreman J., Lee O., Trznadel M., David A., Kudoh T., Tyler C.R. (2017). Acute Toxicity, Teratogenic, and Estrogenic Effects of Bisphenol A and Its Alternative Replacements Bisphenol S, Bisphenol F, and Bisphenol AF in Zebrafish Embryo-Larvae. Environ. Sci. Technol..

[69] Arancio A.L., Cole K.D., Dominguez A.R., Cohenour. E.R., Kadie J., Maloney W.C., Schuh S.M. (2018). Bisphenol A, Bisphenol AF, di-n-butyl phthalate, and 17β-Estradiol have shared and unique dose-Dependent effects on early embryo cleavage divisions and development in *Xenopus laevis*. Reprod. Toxicol..

[70] Arancio A.L., Cole K.D., Dominguez A.R., Cohenour. E.R., Kadie J., Maloney W.C., Schuh S.M. (2019). Data demonstrating distinct embryonic developmental defects induced by bisphenol A alternatives. Data Brief.

[71] Harnett K.G., Chin A., Schuh S.M. (2021). BPA and BPA alternatives BPS, BPAF, and TMBPF, induce cytotoxicity and apoptosis in rat and human stem cells. Ecotoxicol. Environ. Saf..

[72] Rosenmai A.K., Dybdahl M., Pederson M., van Vugt-Lussenburg B.M.A., Wedebye E.B., Taxvig C., Vinggaard A.M. (2014). Are structural analogues to bisphenol A safe alternatives?. Toxicol. Sci..

[73] Vandenberg L.N., Hunt P.A., Gore A.C. (2019). Endocrine disruptors and the future of toxicology testing—Lessons from CLARITY–BPA. Nat. Rev. Endocrinol..

[74] Hunt P.A., Koehler K.E., Susiarjo M., Hodges C.A., Ilagan A., Voigt R.C., Thomas S., Thomas B.F., Hassold T.J. (2003). Bisphenol a exposure causes meiotic aneuploidy in the female mouse. Curr. Biol..

[75] Kojima H., Takeuchi S., Sanoh S., Okuda K., Kitamura S., Uramaru N., Sugihara K., Yoshinari K. (2019). Profiling of bisphenol A and eight of its analogues on transcriptional activity via human nuclear receptors. Toxicology.

[76] Liang S., Yin L., Yu K.S., Hofmann M.-C., Yu X. (2016). High-Content Analysis Provides Mechanistic Insights into the Testicular Toxicity of Bisphenol A and Selected Analogues in Mouse Spermatogonial Cells. Toxicol. Sci..

[77] Michałowicz J., Mokra K., Bąk A. (2015). Bisphenol A and its analogs induce morphological and biochemical alterations in human peripheral blood mononuclear cells (In Vitro study). Toxicol. In Vitro.

[78] Wang C., Zhang J., Li Q., Zhang T., Deng Z., Lian J., Jia D., Li R., Zheng T., Ding X. (2017). Low concentration of BPA induces mice spermatocytes apoptosis via GPR30. Oncotarget.

[79] Wu M., Pan C., Chen Z., Jiang L., Lei P., Yang M. (2017). Bioconcentration pattern and induced apoptosis of bisphenol A in zebrafish embryos at environmentally relevant concentrations. Environ. Sci. Pollut. Res..

[80] Boucher J.G., Ahmed S., Atlas E. (2016). Bisphenol S Induces Adipogenesis in Primary Human Preadipocytes from Female Donors. Endocrinology.

[81] Pelch K., Wignall J.A., Goldstone A.E., Ross P.K., Blain R.B., Shapiro A.J., Holmgren S.D., Hsieh J.-H., Svoboda D., Auerbach S.S. (2019). A scoping review of the health and toxicological activity of bisphenol A (BPA) structural analogues and functional alternatives. Toxicology.

[82] Sun X., Zhu Y., Yin H.Y., Guo Z.Y., Xu F., Xiao B., Jiang W.L., Guo W.M., Meng H.Y., Lu S.B. (2018). Differentiation of adipose-derived stem cells into Schwann cell-like cells through intermittent induction: Potential advantage of cellular transient memory function. Stem Cell Res. Ther..

[83] Peshdary V., Styles G., Gagné R., Yauk C.L., Sorisky A., Atlas E. (2020). Depot-Specific Analysis of Human Adipose Cells and Their Responses to Bisphenol S. Endocrinology.

[84] Sargis R.M., Johnson D.N., Choudhury R.A., Brady M.J. (2010). Environmental Endocrine Disruptors Promote Adipogenesis in the 3T3-L1 Cell Line through Glucocorticoid Receptor Activation. Obesity.

[85] De Filippis E., Li T., Rosen E.D. (2018). Exposure of adipocytes to bisphenol-A in vitro interferes with insulin action without enhancing adipogenesis. PLoS ONE.

[86] Yang Y., Lu L., Zhang J., Yang Y., Wu Y., Shao B. (2014). Simultaneous determination of seven bisphenols in environmental water and solid samples by liquid chromatography–electrospray tandem mass spectrometry. J. Chromatogr. A.

[87] Song S., Ruan T., Wang T., Liu R., Jiang G. (2012). Distribution and Preliminary Exposure Assessment of Bisphenol AF (BPAF) in Various Environmental Matrices around a Manufacturing Plant in China. Environ. Sci. Technol..

[88] Chernis N., Masschelin P., Cox A.R., Hartig S.M. (2020). Bisphenol AF promotes inflammation in human white adipocytes. Am. J. Physiol. Cell Physiol..

[89] Skledar D.G., Carino A., Trontelj J., Troberg J., Distrutti E., Marchianò S., Tomašič T., Zega A., Finel M., Fiorucci S. (2019). Endocrine activities and adipogenic effects of bisphenol AF and its main metabolite. Chemosphere.

[90] Cornwall W. (2020). To replace controversial plastic additive BPA, a chemical company teams up with unlikely allies. Science.

[91] Szafran A.T., Stossi F., Mancini M.G., Walker C.L., Mancini M.A. (2017). Characterizing properties of non-estrogenic substituted bisphenol analogs using high throughput microscopy and image analysis. PLoS ONE.

