# Supplementary material for: BPA, BPAF and TMBPF Alter Adipogenesis and Fat Accumulation in Human Mesenchymal Stem Cells, with Implications for Obesity
Source: Int J Mol Sci. 2021 May 19;22(10):5363. doi: 10.3390/ijms22105363 (PMC8160667; doi:10.3390/ijms22105363)
Supplement: Supplementary file 1 [file ijms-22-05363-s001.zip › ijms-1188072-supplementary.pdf]

BPA, BPAF, and TMBPF alter adipogenesis and fat accumulation in human mesenchymal stem cells, with implications for obesity

Isabel C. Cohen, Emry R. Cohenour, Kristen G. Harnett, and Sonya M. Schuh

Supplemental Figures

Figure S1

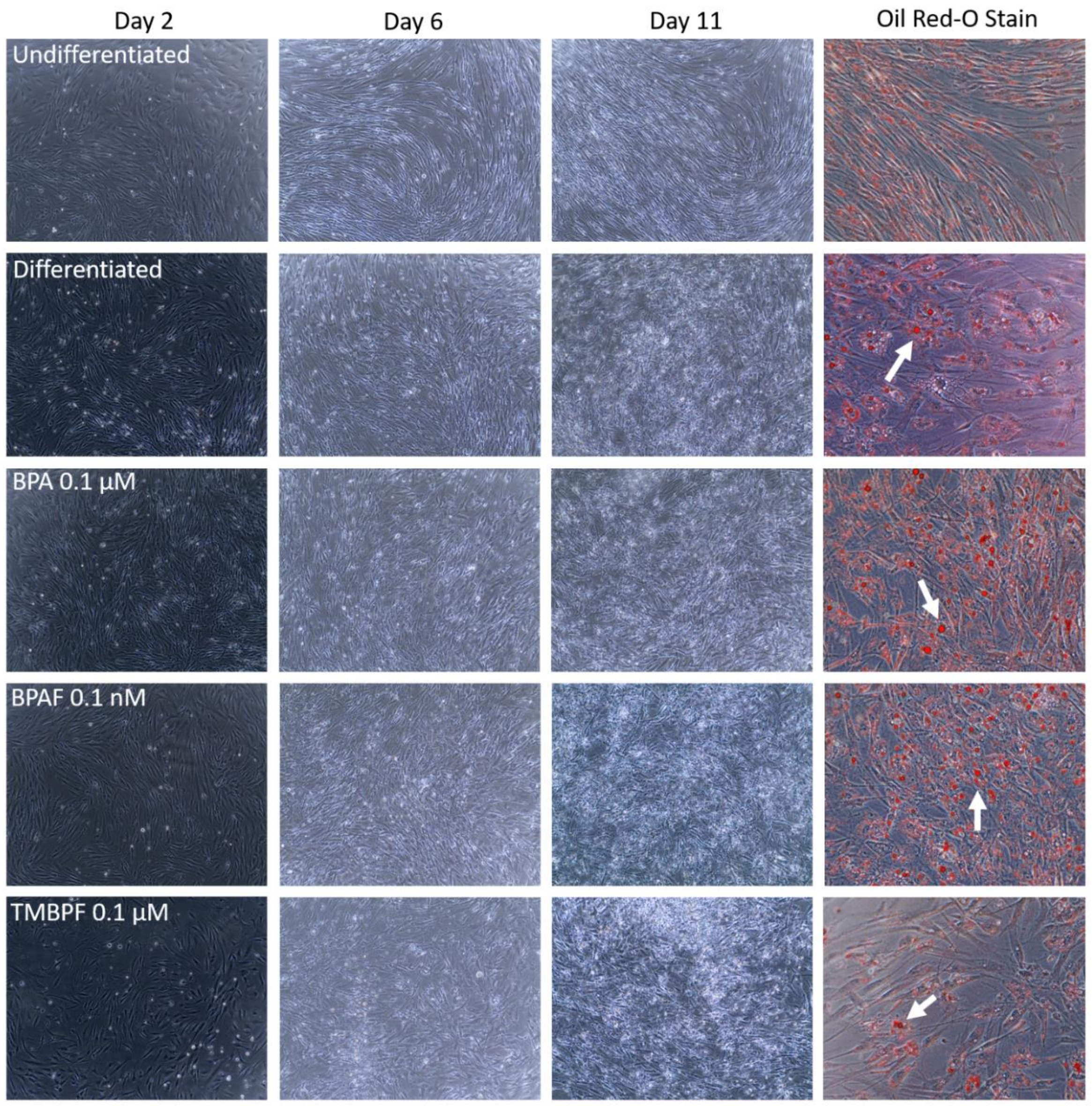

Figure S1. BPA and BPAF increase adipogenesis during the differentiation process. Brightfield images of undifferentiated cells, control differentiated cells, and cells treated with either 0.1 μM BPA, 0.1 nM BPAF, or 0.1 μM TMBPF on Day 2, 6 and 11 during the adipocyte differentiation process. Notice the greater numbers of adipocytes in BPA- and BPAF-treated cells, and the fewer numbers of adipocytes and lipid vacuoles in the TMBPF-treated cells. Arrows indicate lipid vacuoles.

Figure S2

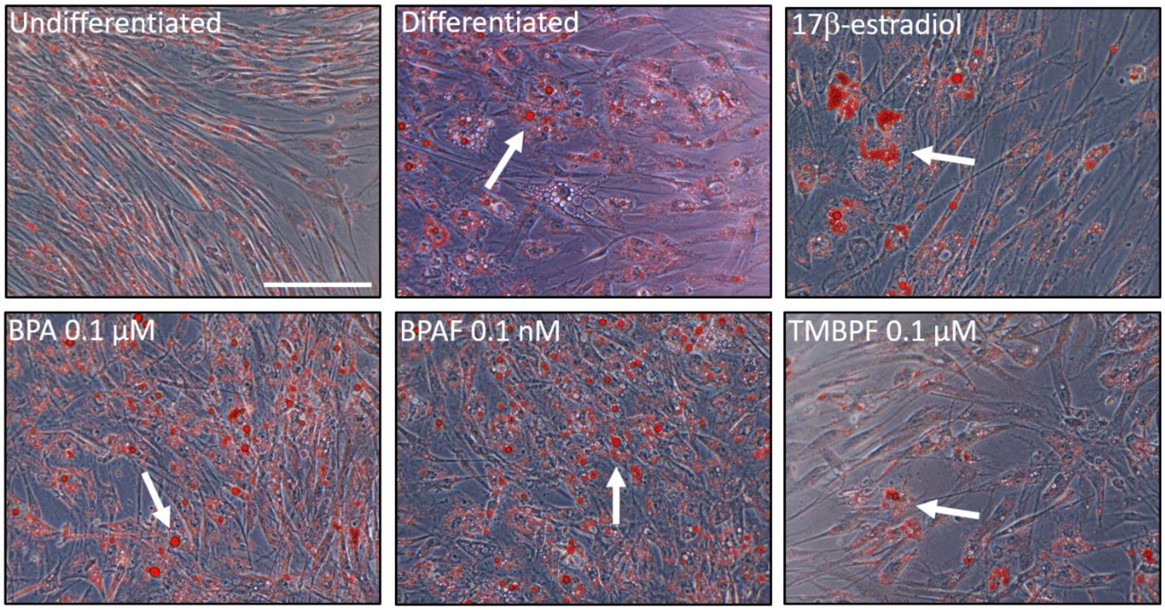

Figure S2. BPA and BPAF increase adipogenesis. Brightfield images of undifferentiated cells, control differentiated cells, or cells treated with either 10 µM 17β-estradiol, 0.1 µM BPA, 0.1 nM BPAF, or 0.1 µM TMBPF during the adipocyte differentiation process. Notice the relatively higher level of adipogenesis and lipid production in the BPA- and BPAF-differentiated cells (see red spheres; white arrows indicate large lipid vacuoles) (200X magnification; scale bar = 150 µm).

Figure S3

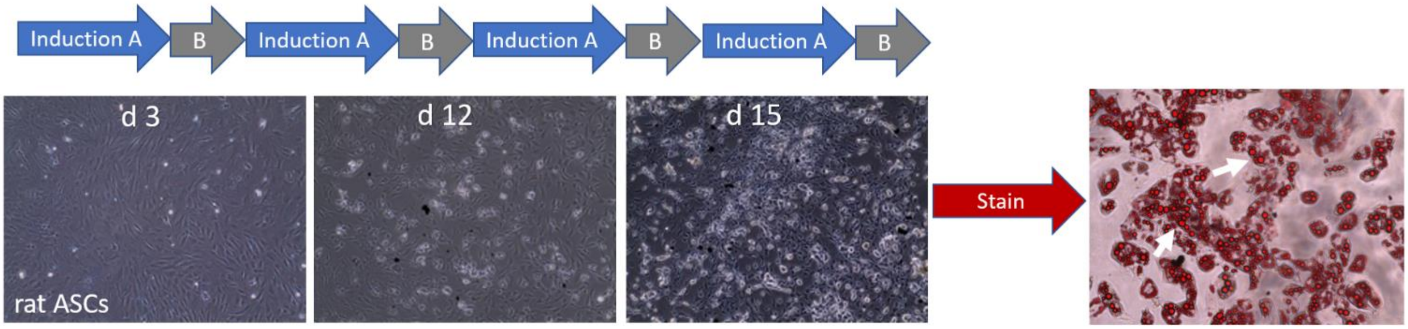

Figure S3. The differentiation process of rat adipose-derived stem cells into adipocytes. Brightfield images of cells treated with induction medium A for 3 days, then maintenance medium B for 24 hours, for 4 cycles. All cells were fixed, stained with Oil Red-O, imaged, and quantified for lipid vacuoles (white arrows).
